# Metabolic investigation of host/pathogen interaction using MS2-infected *Escherichia coli*

**DOI:** 10.1186/1752-0509-3-121

**Published:** 2009-12-30

**Authors:** Rishi Jain, Ranjan Srivastava

**Affiliations:** 1 Department of Chemical, Materials and Biomolecular Engineering, University of Connecticut, Storrs, CT 06269, USA; 2 Biosciences Division, Oak Ridge National Laboratory, Mail Stop 6038, Oak Ridge, TN 37831, USA

## Abstract

**Background:**

RNA viruses are responsible for a variety of illnesses among people, including but not limited to the common cold, the flu, HIV, and ebola. Developing new drugs and new strategies for treating diseases caused by these viruses can be an expensive and time-consuming process. Mathematical modeling may be used to elucidate host-pathogen interactions and highlight potential targets for drug development, as well providing the basis for optimizing patient treatment strategies. The purpose of this work was to determine whether a genome-scale modeling approach could be used to understand how metabolism is impacted by the host-pathogen interaction during a viral infection. *Escherichia coli*/MS2 was used as the host-pathogen model system as MS2 is easy to work with, harmless to humans, but shares many features with eukaryotic viruses. In addition, the genome-scale metabolic model of *E. coli *is the most comprehensive model at this time.

**Results:**

Employing a metabolic modeling strategy known as "flux balance analysis" coupled with experimental studies, we were able to predict how viral infection would alter bacterial metabolism. Based on our simulations, we predicted that cell growth and biosynthesis of the cell wall would be halted. Furthermore, we predicted a substantial increase in metabolic activity of the pentose phosphate pathway as a means to enhance viral biosynthesis, while a break down in the citric acid cycle was predicted. Also, no changes were predicted in the glycolytic pathway.

**Conclusions:**

Through our approach, we have developed a technique of modeling virus-infected host metabolism and have investigated the metabolic effects of viral infection. These studies may provide insight into how to design better drugs. They also illustrate the potential of extending such metabolic analysis to higher order organisms, including humans.

## Background

Viruses are the cause of a variety of diseases ranging from the mildly annoying common cold to the frighteningly lethal ebola. Viruses infect their hosts and hijack the host machinery, using it to produce more progeny viral particles. Viruses, being obligate parasites, require host resources to replicate. Therefore, viral infections lead to alterations in the metabolism of the host, shifting in favor of viral protein production. A systems biology approach for studying these metabolic changes in the host cell could provide new insights to potential drug targets [1], motivating this study.

Systems biology deals with the studying of organisms that are viewed as one collaborative network of genes, proteins and other metabolites. Recent advancements in high-throughput experimental techniques have brought a flood of information in the form of genomic, transcriptomic, proteomic and metabolomic datasets. Systems biology is answering the growing need for the integration and interpretation of these heterogeneous datasets. One of the methodologies of systems biology, a constraints-based modeling approach [2], has been successfully demonstrated in identifying potential drug targets [3]. Genome-scale metabolic models of disease-causing organisms have been constructed and evaluated to identify potential drug targets [3-5].

In this study, we have demonstrated the application of the constraints-based, flux balance analysis approach to investigate the effects of host-pathogen interactions on host metabolism. The pathogen-host pair under consideration was a bacterial virus, MS2, and its host, *Escherichia coli *C-3000. MS2 is a lytic RNA bacteriophage, belonging to the Leviviridae family of viruses and infects F+ *Escherichia coli *cells [6].

The *Escherichia coli*/MS2 system was chosen for a number of reasons. MS2 is harmless to humans and yet possesses many of the same features as its eukaryotic-infecting viral cousins, and as a result may aid in our understanding of RNA viruses in general. It can be cultured quickly, cheaply, and safely, making it easy to work with. Furthermore, the genome-scale metabolic model of *E. coli *is the most exhaustive one to date. These factors combine to make the *E. coli/*MS2 model system ideal for such a study.

The constraints-based modeling approach was used to describe only a part of the infection process. Based on an experimental study tracking the macromolecular synthesis in MS2-infected *Escherichia coli *[7], the infection process can be divided into 3 parts - an early transient period where the infection process is initiated, a middle steady state period where the viral protein synthesis has displaced the host protein synthesis and a late transient period where all biosynthesis has diminished and lysis is approaching. This study used the constraints-based modeling approach to investigate the middle steady state period only.

The *Escherichia coli *genome-scale metabolic model, iAF1260 [8] was used as a basis to represent both - the uninfected cells and the infected cells. C-3000, the *Escherichia coli *strain used in this study and MG1655, the *Escherichia coli *strain used to model iAF1260 are both *Escherichia coli *K-12 derivatives. As a result, iAF1260 was used to represent the uninfected cells. The genome-scale metabolic model of MS2-infected *E. coli *was based on iAF1260, but was modified to account for the viral infection process. The constraints for these models such as the glucose uptake rate, the oxygen uptake rate and the growth rates of the cells were measured experimentally. Finally, the genome scale metabolomes of the infected and the uninfected cells were compared to find what parts of the metabolic network were activated, upregulated, downregulated or deactivated.

## Methods

For all *in silico *reconstructions, linear programming was carried out using the GLPSOL (version 4.38) solver, which is available as a part of the GNU Linear Programming Kit [9]. In addition, to capture the effects of alternate optimal solutions, flux variability analysis [10] was carried out for all *in silico *reconstructions.

### *In silico *reconstruction of genome-scale metabolic network of uninfected *Escherichia coli*

The reconstruction of the uninfected host metabolism was based on the model iAF1260 [8]. In brief, the system was defined as the metabolic network that included extracellular, cytoplasmic and periplasmic metabolites. Exchange reactions were responsible for metabolites entering or leaving the system. Transport reactions accounted for the movement of metabolites across the inner and outer membranes. The remaining reactions accounted for the biochemical interactions among metabolites.

The constructed stoichiometric matrix () of dimensions *m *× *n *represented *m *number of metabolites and *n *number of reactions. Each element of the matrix, *S*_*ij*_, represented the stoichiometric coefficient of the *i*^*th *^metabolite in the *j*^*th *^reaction. Flux balance analysis [2,11,12] was used to determine the steady state flux distribution of the metabolic network, mathematically represented by(1)

where, *v *represented a vector of reaction fluxes. An optimization technique, linear programming, was applied to find a specific solution to the underdetermined linear problem. Additional constraints in the form of bounds were placed on each of the fluxes. For all the irreversible reactions, the limits were 0 ≤ *v*_*i *_≤ ∞ and for the reversible reactions, the limits were -∞ ≤ *v*_*i *_≤ ∞. For the exchange fluxes, the limits of 0 ≤ *v*_*i *_≤ ∞ were applied for the metabolites that were not present in the growth medium and the limits of -∞ ≤ *v*_*i *_≤ ∞ for the metabolites that were provided by the growth medium. In addition, the fluxes of the limiting reactions were constrained to the experimentally measured values.

Based on studies that have systematically evaluated various objective functions [13-15] on experimental systems similar to the one used in this study, maximization of ATP yield was used to model the uninfected cells. Maximization of ATP yield implies the maximization of the accumulation of ATP in the metabolic network. The biological rationale behind this approach is to account for the requirement of ATP in the processes that are not included in the metabolic model, such as mobility, futile cycles [16] and heat generation. The allowance of accumulation of ATP in the metabolic network would seem to be in contradiction with the basic assumption of steady state of metabolic networks, which implies that there is no accumulation of metabolites. This issue was easily reconciled by including an exchange reaction flux to represent ATP requirements not directly accounted for in the metabolic network as defined. Therefore, the objective function was defined as the maximization of this exchange reaction.

### *In silico *reconstruction of genome-scale metabolic network of infected *Escherichia coli*

To incorporate the effects of the bacteriophage infection, a new subset of metabolites corresponding to the viral proteins and the viral genome was added to the uninfected model. The MS2 bacteriophage encodes for four proteins - coat protein, maturation protein, replicase protein and lysis protein. The MS2 bacteriophage genome is a positive strand of RNA. The positive viral strands are used as templates by the replicase protein to produce negative strands that further serve as templates to produce positive viral strands [17]. As a result, the new subset of metabolites consisted of the four viral proteins, positive viral RNA and negative viral RNA. Reactions corresponding to the formation of these viral metabolites from the host metabolites were defined. In each of these reactions the reactants were amino acids producing viral proteins or nucleotides producing positive or negative viral RNA strands. The stoichiometry of these reactions was based on the amino acid sequence of the viral proteins or the nucleotide sequence of the MS2 genome. Exchange reactions that transferred the viral metabolites out of the host system were also included. A list of viral reactions and metabolites are presented in Table 1.

**Table 1:** List of viral reactions and metabolites.

Name	Description
**Metabolic Reactions**	
viralA	(35) ala-L + (29) arg-L + (17) asn-L + (13) asp-L + (3) cys-L + (18) gln-L + (17) glu-L + (28) gly + (5) his-L + (16) ile-L + (36) leu-L + (14) lys-L + (8) met-L + (16) phe-L + (17) pro-L + (36) ser-L + (27) thr-L + (12) trp-L + (16) tyr-L + (30) val-L → mat_v
viralC	(14) ala-L + (4) arg-L + (10) asn-L + (4) asp-L + (2) cys-L + (6) gln-L + (5) glu-L + (9) gly + (8) ile-L + (7) leu-L + (6) lys-L + (3) met-L + (4) phe-L + (6) pro-L + (13) ser-L + (9) thr-L + (2) trp-L + (4) tyr-L + (14) val-L → coat_v
viralL	(3) ala-L + (8) arg-L + (2) asn-L + asp-L + cys-L + (9) gln-L + (3) glu-L + his-L + (3) ile-L + (12) leu-L + (2) lys-L + met-L + (5) phe-L + (4) pro-L + (6) ser-L + (9) thr-L + (2) tyr-L + (3) val-L → lys_v
viralR	(41) ala-L + (42) arg-L + (18) asn-L + (33) asp-L + (7) cys-L + (15) gln-L + (22) glu-L + (44) gly + (10) his-L + (32) ile-L + (50) leu-L + (25) lys-L + (10) met-L + (28) phe-L + (27) pro- L + (48) ser-L + (31) thr-L + (9) trp-L + (21) tyr-L + (32) val- L → rep_v
viralposRNA	(834) atp + (933) ctp + (927) gtp + (875) utp → posRNA_v
viralnegRNA	(875) atp + (927) ctp + (933) gtp + (834) utp → negRNA_v

**Exchange Reactions**	
EXviralA	mat_v ↔
EXviralC	coat_v ↔
EXviralL	lys_v ↔
EXviralR	rep_v ↔
EXviralposRNA	posRNA_v ↔
EXviralnegRNA	negRNA_v ↔

**Viral Metabolites**	
mat_v	viral maturation protein
coat_v	viral coat protein
Lys_v	viral lysis protein
rep_v	viral replicase protein
posRNA_v	positive sense viral RNA genome
negRNA_v	negative sense viral RNA genome

The regulated expression of viral genes was incorporated in the infected cell model. This was achieved by fixing the ratios of the fluxes of maturation protein production, replicase protein production and lysis protein production to coat protein production. The coat gene is expressed constitutively and forms the major fraction of the viral proteins that are produced [6,18,19]. The other viral proteins are produced in much smaller amounts due to the regulated expression of their corresponding genes [6]. Previous studies have shown that the amounts of each of the maturation and replicase proteins were only 1/10^th ^of that of the coat protein [18,19]. As for the lysis protein, the amount was found to be only 1/25^th ^of that of the coat protein [20]. These ratios were used in the model to account for the regulated expression of the viral genes. The ratios of fluxes corresponding to viral maturation protein production, viral replicase protein production and viral lysis protein production to viral coat protein production were fixed to 1:10, 1:10 and 1:25, respectively.

Replication of the viral genome was also integrated into the infected cell model by including the positive and negative viral RNA strands as metabolites and the corresponding reactions that produce these metabolites. However, to constrain the fluxes of these reactions, there has been no experimental quantification of the number of positive and negative strands per cell. Hence, the fluxes of the production of these positive and negative strands were constrained based on the composition of MS2 virions. Each MS2 virion consists of 180 copies of the coat protein and one copy of the A protein and a positive strand of the viral genome [6]. Therefore, the ratio of each of the fluxes of positive and negative viral genomic RNA production to the coat protein production was fixed to 1:180.

In addition to the application of constraints of the uninfected model, the exchange flux corresponding to the glucose uptake rate was constrained to the experimentally measured value of 12 mmol/g DW h. The growth rate and the oxygen uptake rate of the host were bound by the limits of 0 ≤ *v*_*i *_≤ ∞ and -∞ ≤ *v*_*i *_≤ ∞, respectively. Finally, appropriate objective functions were applied.

The most significant effect of the infection process is the change in the objective of the metabolic network. The objective function used to model the MS2 infected *Escherichia coli *cells was to maximize the viral reaction fluxes. The underlying reasoning for this approach was that the virus is a parasite and takes over the machinery of its host to produce viral components. Viral protein synthesis dominates over the host protein synthesis [7]. Other objective functions considered were the simultaneous maximization of ATP yield and the viral fluxes, as well as the simultaneous maximization of growth rate and the viral fluxes. These objective functions resulted in no viral protein production and therefore, were not considered further in this study. This result reiterates the fact that host protein synthesis has to be eclipsed for viral protein synthesis to prevail.

### Experimental methods

The experimental system consisted of the bacteriophage MS2 (ATCC 15597-B1) and its host *Escherichia coli *C-3000 (ATCC 15597) in minimal M9 media [21] (supplemented with 2 mM CaCl_2 _and 10 μg/ml thiamine). The experimental methods used in this study were the same as described previously [22]. In brief, for the uninfected cells, C-3000 was grown in minimal M9 media at 37°C (batch culture) and OD_600 _was measured using a Biomate 3 Spectrophotometer (Thermo Spectronic, Rochester, NY), glucose concentrations were measured using a YSI 2700 Select Biochemical Analyzer (YSI, Yellow Springs, OH) and dissolved oxygen was measured using a Dissolved Oxygen Meter SM600 (Milwaukee Instruments, Inc, Rocky Mount, NC).

For the infected cells, a C-3000 culture in minimal M9 media at 37°C (batch culture) was infected with MS2 phage at an OD_600 _of 0.8 and an MOI of 10. OD_600 _and glucose concentrations were measured. In addition, two kinds of plaque assays were carried out as described in previously published work [22]. The first method was used to quantify the combined sum of infected cells and free phages and started with the mixing of 100 μl of an appropriately diluted sample of the culture with 3 ml E medium (10 g/l of tryptone, 1 g/l of yeast extract and 8 g/l of NaCl; also referred to as "EM") soft agar and 100 μl of the plating culture (an overnight culture of *Escherichia coli *C-3000). This mixture was poured on pre-warmed EM plates and incubated overnight at 37°C. Plaques were counted the next day. The second method was used to quantify free phages only and was similar to the above technique. The only differences were the use of *Escherichia coli *C-3000+pBAD102 (ampicillin resistant) as the plating culture and the addition of 100 g/ml ampicillin to EM soft agar and EM plates. The presence of ampicillin did not allow the infected cells to grow as they were ampicillin sensitive. As a result, the plaques that were formed were only due to the free phage. Utilizing both approaches allowed for determination of free phage density and infected cell density.

### Determination of glucose and oxygen uptake rates of uninfected *Escherichia coli *C-3000 cells

Changes in the glucose concentration of the medium were used to determine the glucose uptake rate of uninfected *Escherichia coli *C-3000 cells in minimal M9 media. Cell density and glucose concentration data were fitted to the following set of differential equations -(2)

where X is the cell density, S is the glucose concentration, μ is the growth rate of the cells and k_glu _is the glucose uptake rate of the cells.

Similarly, oxygen uptake rate of uninfected *Escherichia coli *C-3000 cells in minimal M9 media was determined on the basis of changes in the dissolved oxygen concentrations. Cell density and dissolved oxygen concentration data was fitted to the following set of differential equations -(4)

where X is the cell density, μ is the growth rate of the cells, S_oxy _is the dissolved oxygen concentration, k_L_a is the mass transfer coefficient,  is the saturated oxygen concentration in the medium and k_oxy _is the oxygen uptake rate of the cells. The first term on the right hand side of (5) relates to the transfer of atmospheric oxygen into the medium.

### Determination of glucose uptake rate of MS2 infected *Escherichia coli *C-3000 cells

Glucose uptake rate of MS2 infected cells was determined indirectly with the combination of experimental data and a mathematical model describing the phage-host dynamics. The technique used in this study is similar to the one presented previously [22] with minor modifications described below. Specifically in this version, glucose uptake rate by infected cells was included.(6)

where X is uninfected cell density, Z is phage-sensitive uninfected cell density, R is phage-resistant uninfected cell density, Y is phage-infected cell density, P is free phage density and S is substrate (glucose) concentration.

The Berkeley Madonna software package (version 8.3.14) [23] was used to estimate parameters in the determination of glucose and oxygen uptake rates of uninfected cells and glucose uptake rate of infected cells.

## Results and Discussion

### Experimental results

The growth rate of the uninfected *Escherichia coli *C-3000 in minimal M9 media was found to be 0.65 ± 0.02 h^-1 ^(n = 5). The glucose uptake rate of the uninfected cells was 12 ± 0.5 mmol/(g DW h) (n = 3) and the oxygen uptake rate was 27 ± 1 mmol/(g DW h) (n = 2). The glucose uptake rate of the MS2-infected *Escherichia coli *C-3000 cells in minimal M9 media was the same as that of the uninfected cells, 12 ± 1 mmol/(g DW h) (n = 3). See Additional file 1 for the original data that was used to determine the above mentioned cell properties.

### Infected *Escherichia coli *model

The results of the genome-scale model of the infected cell predicted the flux of the viral coat protein production to be 4.12 × 10^-2 ^mmol/g DW h and the flux variability analysis showed no variation in this value. The experimentally calculated value of the flux, 1.05 × 10^-5 ^mmol/g DW h, was based on the burst size of 10 ± 1 pfu/cell [22], latent period of 60 ± 2 min [22] and the composition of the viral capsid containing 180 copies of the coat protein. The discrepancy between the experimentally calculated value and the model prediction may be explained by the fact that the experimentally calculated value only accounts for the plaque forming units (pfu). It does not account for the defective MS2 viral particles that are also produced in host cells [24-27] and are incapable of forming plaques. Although, no literature was found regarding the particle to pfu ratio of MS2, studies have reported particle to pfu ratios of bacteriophage f2, very closely related to MS2, that range from 7 to 39 [28,29]. In addition, the experimentally measured burst size assumed that all infected cells produced plaque forming units and an average number of progeny virus particles were calculated on a per cell basis. In contrast, studies specific to MS2 bacteriophage and the use of minimal media have reported that the fraction of infected cells producing plaque forming units (infective phages) is as low as 0.01 [24,30,31]. This suggests that the average burst size may further be increased by as much as two orders of magnitude. Based on these findings, overall, the burst size could be increased by three orders of magnitude increasing the experimentally calculated value of the coat protein production flux by three orders of magnitude. This may account for the differences between the experimentally measured value and the model prediction of coat protein production flux.

### Comparison of the infected and uninfected cell metabolomes

A comparison of the major pathways in the infected cell metabolome was made with those in the uninfected cell metabolome. Flux variability analysis showed no variability in the fluxes of the pathways discussed below - both in the uninfected and the infected models.

The infected cell model predicted that the host cell does not grow. The value of the flux corresponding to the biomass production was zero, which is consistent with the experimental observations of MS2/C-3000 viral dynamics [22]. It was also noted that all the cell wall synthesis and membrane lipid metabolism reactions were deactivated. This result also contributes to the fact that the biomass growth rate was zero and this weakening of the cell wall may further aid the virus in the lysis of the host at the end of the infection cycle.

**Figure 1:**
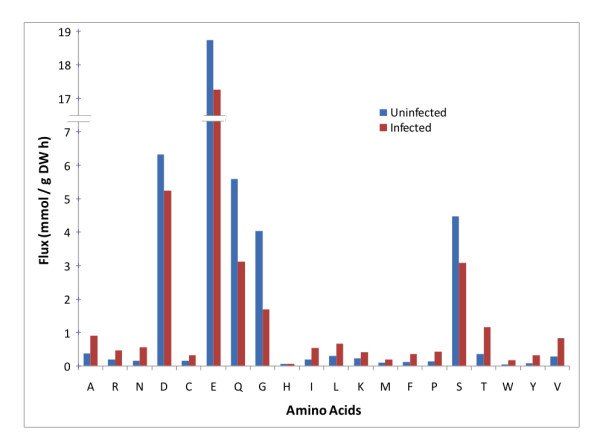
**Comparison of the biosynthesis of amino acids in a MS2 phage-infected *Escherichia coli *cell with an uninfected *Escherichia coli *cell**. Little change to a five-fold increase was seen although aspartate (D), glutamine (Q), glycine (G) and serine (S) biosynthesis rates decreased by as much as two-fold. The x-axis labels represent the single letter amino acid abbreviations, a key for which is available at http://en.wikipedia.org/wiki/Amino_acid. Figure legend text.

**Figure 2:**
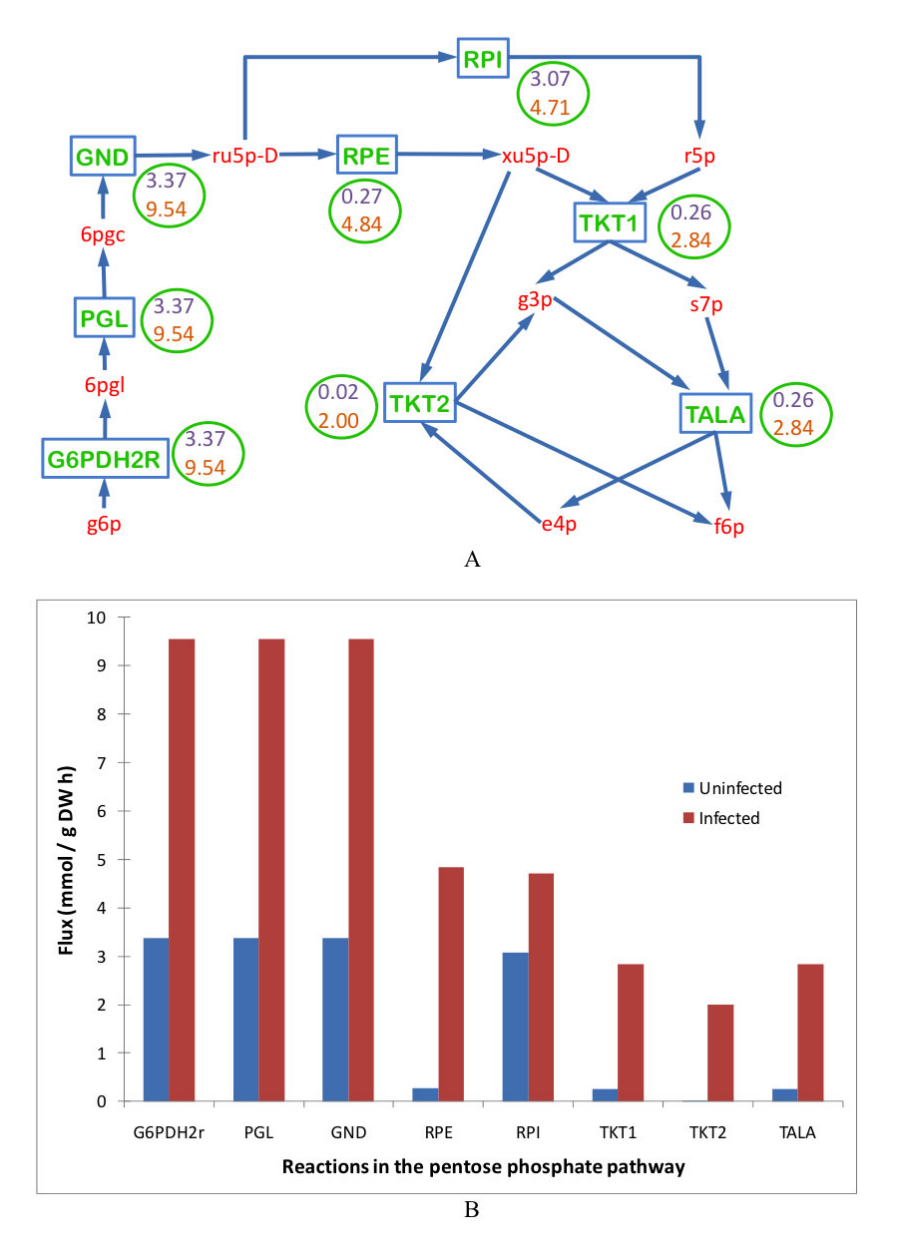
**Comparison of the pentose phosphate pathway in an uninfected *Escherichia coli *cell and a MS2 phage-infected *Escherichia coli *cell**. Relative to uninfected cells, all the reactions involved in the pentose phosphate pathway were upregulated during infection. (A) Schematic representation of the pentose phosphate pathway. Words in red represent metabolites. Rectangular boxes represent reactions, the key for which is provided in the Table 2. The numbers in the circle represent the flux values of the corresponding reactions in uninfected cell (top value) and infected cell (bottom value). (B) Quantitative representation of variation in the uninfected and infected cell fluxes of the pentose phosphate pathway. Key to the x-axis labels is given in the Table 2.

All metabolic resources were diverted to the biosynthesis of amino acids in the infected cells instead of the production of biomass as in the uninfected cells. Overall, there was anywhere from little change to a five-fold increase in the biosynthesis of amino acids except for aspartate (D), glutamine (Q), glycine (G) and serine (S) synthesis rates as shown in Figure 1. Biosynthesis of aspartate, glutamine, glycine and serine was downregulated as, in addition to protein synthesis, these amino acids also took part in the ATP biosynthesis that by itself was downregulated as a result of change in the objective of the metabolic network. Also, the pentose phosphate pathway was upregulated from anywhere between two-fold to 100-fold as shown in Figure 2.

**Table 2:** List of bacterial reactions and metabolites.

Name	Description
**Reactions**	
**Pentose Phosphate Pathway (Figure 2)**	
G6PDH2r	g6p + nadp → 6pgl + h + nadph
PGL	6pgl + h2o → 6pgc + h
GND	6pgc + nadp → co2 + nadph + ru5p-D
RPE	ru5p-D → xu5p-D
RPI	ru5p-D → r5p
TKT1	r5p + xu5p-D → g3p + s7p
TKT2	e4p + xu5p-D → f6p + g3p
TALA	g3p + s7p → e4p + f6p

**Glycolytic Pathway (Figure 3)**	
GLCptspp	glc-D + pep → g6p + pyr
HEX1	glc-D + atp → g6p + adp + h
PGI	g6p → f6p
PFK	atp + f6p → adp + fdp + h
FBA	fdp → dhap + g3p
GAPD	g3p + nad + pi → 13dpg + h + nadh
PGK	13dpg + adp → 3pg + atp
PGM	3pg → 2pg
ENO	2pg → h2o + pep

**TCA Cycle (Figure 4)**	
CS	accoa + h2o + oaa → cit + coa + h
ACONTa	cit → acon-C + h2o
ACONTb	acon-C + h2o → icit
ICDHyr	icit + nadp → akg + co2 + nadph
AKGDH	akg + coa + nad → co2 + nadh + succoa
SUCOAS	adp + pi + succoa → atp + coa + succ
SUCDi	q8 + succ → fum + q8h2
FUM	fum + h2o → mal-L
MDH	mal-L + nad → h + nadh + oaa

**Metabolites**	
13dpg	3-Phospho-D-glyceroyl phosphate
2pg	D-Glycerate 2-phosphate
3pg	3-Phospho-D-glycerate
6pgc	6-Phospho-D-gluconate
6pgl	6-phospho-D-glucono-1,5-lactone
Accoa	Acetyl-CoA
acon-C	cis-Aconitate
Adp	Adenosine diphosphate
Akg	2-Oxoglutarate
Atp	Adenosine triphosphate
Cit	Citrate
co2	Carbon dioxide
Coa	Coenzyme A
Dhap	Dihydroxyacetone phosphate
e4p	D-Erythrose 4-phosphate
f6p	D-Fructose 6-phosphate
Fad	Flavin adenine dinucleotide
fadh2	Flavin adenine dinucleotide - reduced
Fdp	D-Fructose 1,6-bisphosphate
Fum	Fumarate
g3p	Glyceraldehyde 3-phosphate
g6p	D-Glucose 6-phosphate
glc-D	D-Glucose
H	H+
h2o	Water
Icit	Isocitrate
mal-L	L-Malate
Nad	Nicotinamide adenine dinucleotide
Nadh	Nicotinamide adenine dinucleotide - reduced
Nadp	Nicotinamide adenine dinucleotide phosphate
Nadph	Nicotinamide adenine dinucleotide phosphate - reduced
Oaa	Oxaloacetate
Pep	Phosphoenolpyruvate
Pi	Phosphate
Pyr	Pyruvate
q8	Ubiquinone-8
q8h2	Ubiquinol-8
r5p	alpha-D-Ribose 5-phosphate
ru5p-D	D-Ribulose 5-phosphate
s7p	Sedoheptulose 7-phosphate
Succ	Succinate
Succoa	Succinyl-CoA
xu5p-D	D-Xylulose 5-phosphate

**Figure 3:**
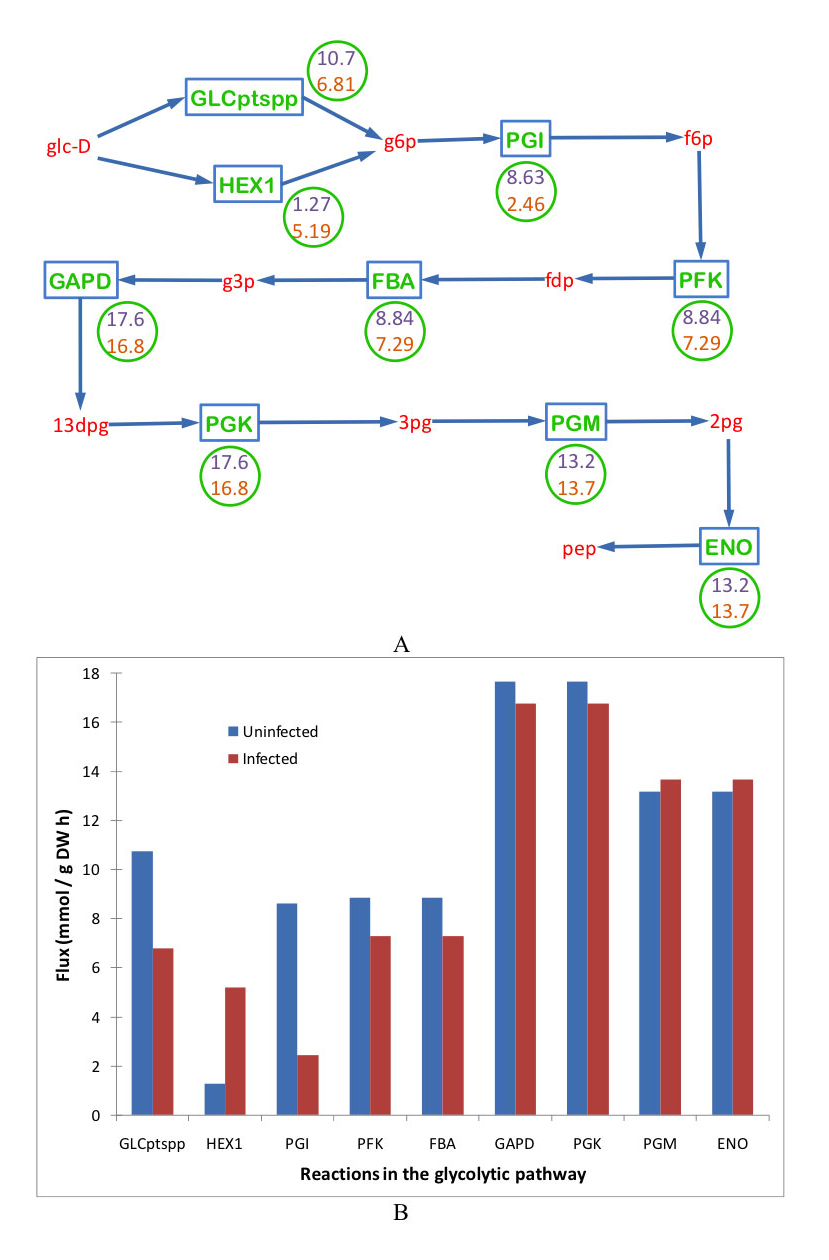
**Comparison of the glycolytic pathway in an uninfected *Escherichia coli *cell and a MS2 phage-infected *Escherichia coli *cell**. All the reactions were unchanged except GLCPTS and HEX1, that correspond to the conversion of glucose to glucose-6-phosphate (g6p) and PGI, that corresponds to the conversion of g6p to fructose-6-phosphate. (A) Schematic representation of the glycolytic pathway. Words in red represent metabolites. Rectangular boxes represent reactions, the key for which is provided in the Table 2. The numbers in the circle represent the flux values of the corresponding reactions in uninfected cell (top value) and infected cell (bottom value). (B) Quantitative representation of variation in the uninfected and infected cell fluxes of the glycolytic pathway. Key to the x-axis labels is given in the Table 2.

**Figure 4:**
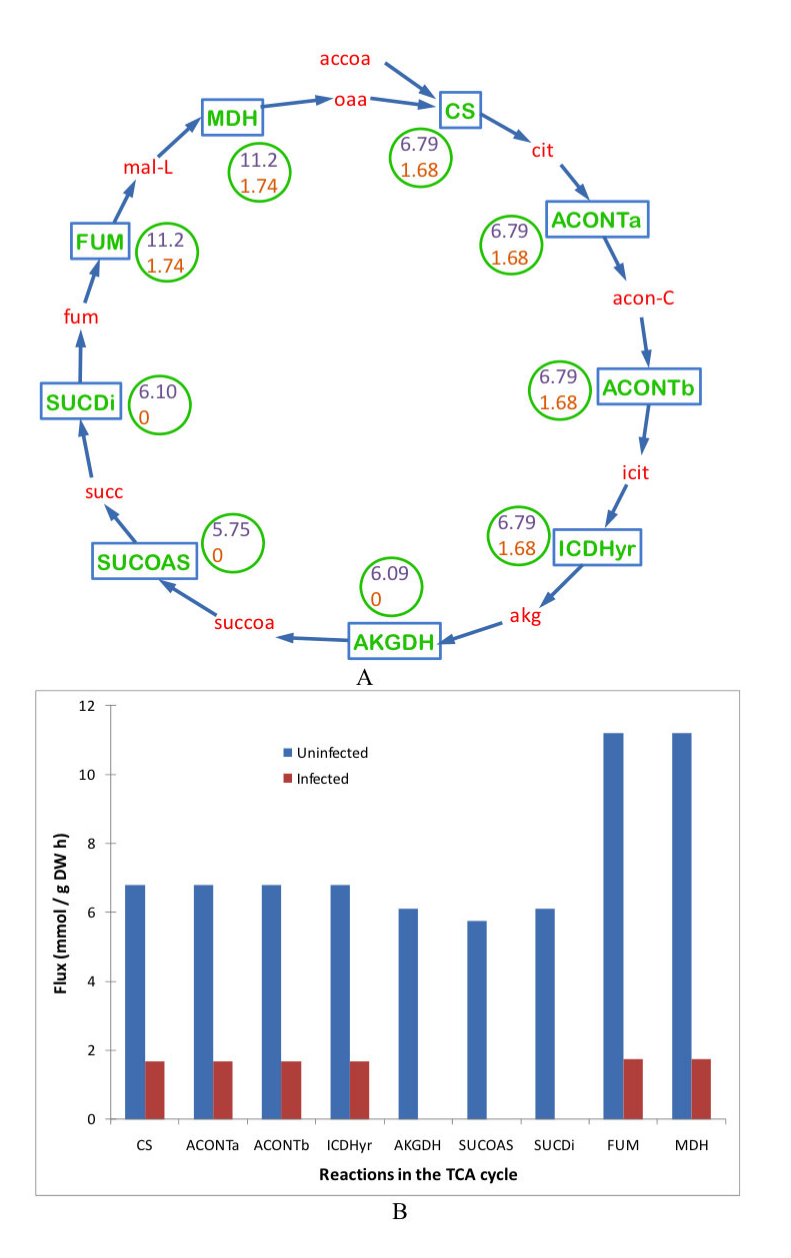
**Comparison of the TCA cycle in a MS2 phage-infected *Escherichia coli *cell with an uninfected *Escherichia coli *cell**. Most of the reactions were downregulated by four to six folds and some of them were even deactivated. (A) Schematic representation of the TCA cycle. Words in red represent metabolites. Rectangular boxes represent reactions, the key for which is provided in the Table 2. The numbers in the circle represent the flux values of the corresponding reactions in uninfected cell (top value) and infected cell (bottom value). (B) Quantitative representation of variation in the uninfected and infected cell fluxes of the TCA cycle. Key to the x-axis labels is given in the Table 2.

The glycolytic pathway remained at almost the same levels as that in the uninfected cells, shown in Figure 3. The only exceptions were a less than two-fold decrease in the flux GLCPTS, four-fold increase in the flux HEX1, both reactions corresponding to the conversion of glucose to glucose-6-phosphate and a three-fold decrease in the flux PGI, corresponding to the conversion of glucose-6-phosphate to fructose-6-phosphate. In contrast, the TCA cycle was severely downregulated, by as much as four to six-fold, as shown in Figure 4. Some of the reactions were completely deactivated.

**Figure 5:**
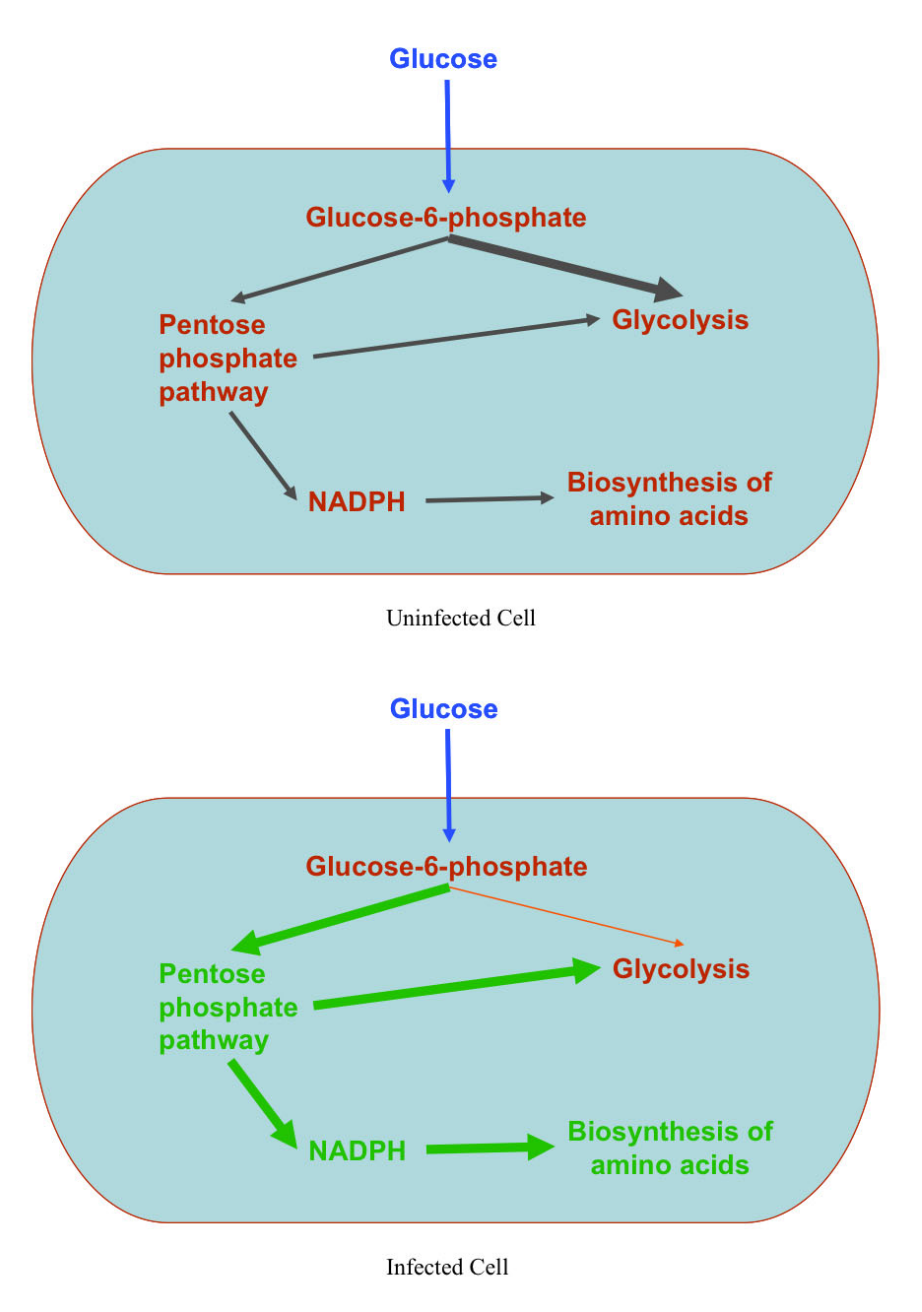
**Comparison of metabolic networks of an uninfected and a MS2 phage-infected *Escherichia coli *cell**. Thick flux arrows represent high reaction fluxes, whereas the thinner flux arrows represent low reaction fluxes. Biosynthesis of amino acids is upregulated in infected cells. NADPH is a pre-cursor that is required in the biosynthesis of most of the amino acids. The pentose phosphate pathway provides NADPH and is therefore upregulated in infected cells. This up-regulation is due to the diversion of a major portion of glucose-6-phosphate (g6p) to the pentose phosphate pathway instead of the glycolytic pathway. The glycolytic pathway is not affected because the loss of g6p is made up by the gain in the amounts of the by-products of the pentose phosphate pathway, fructose-6-phosphate and glyceraldehyde 3-phosphate, which are fed back into the glycolytic pathway.

All of these observations may be explained by linking the metabolic pathways and comparing the metabolic networks of the uninfected and the infected cells, as shown in Figure 5. In an uninfected cell (Figure 5), glucose is taken up and in the process is converted to glucose-6-phosphate through two reactions - the phosphotransferase system reaction (GLCPTS) and the hexokinase reaction. The hexokinase reaction requires ATP as a reagent and is therefore minimized in accordance with the objective function. Major portion of the glucose taken up is used in the GLCPTS reaction. Also, a major portion of the glucose-6-phosphate formed feeds into the glycolytic pathway. However, a minor portion of glucose-6-phosphate is diverted towards the pentose phosphate pathway. Most of the by-products of the pentose phosphate pathway lead back into the glycolytic pathway. In addition, the pentose phosphate pathway is a major contributor to the production of NADPH, a mediator that is required for the biosynthesis of many amino acids.

The changes in the infected cell may be attributed to the up-regulation of biosynthesis of amino acids, as shown in Figure 5. The glucose uptake rates of uninfected and infected cells are similar. A change in the objective function removes the constraints on the usage of ATP that leads to the up-regulation of the hexokinase reaction and the down-regulation of the GLCPTS reaction. However, these changes do not affect the amounts of glucose-6-phosphate, which remains the same in both uninfected and infected cells. The changes start with the division of glucose-6-phosphate. The major portion of glucose-6-phosphate is diverted towards the pentose phosphate pathway, leading to the up-regulation of the pentose phosphate pathway. As a result more NADPH is produced that leads into the up-regulation of biosynthesis of amino acids. Only a minor portion of glucose-6-phosphate feeds the glycolytic pathway leading to the down-regulation of the PGI reaction. Other reactions downstream of the glycolytic pathway are not affected because the up-regulation of the pentose phosphate pathway increases the amounts of the by-products of the pentose phosphate pathway, fructose-6-phosphate and glyceraldehyde 3-phosphate, which are fed back into the glycolytic pathway.

**Figure 6:**
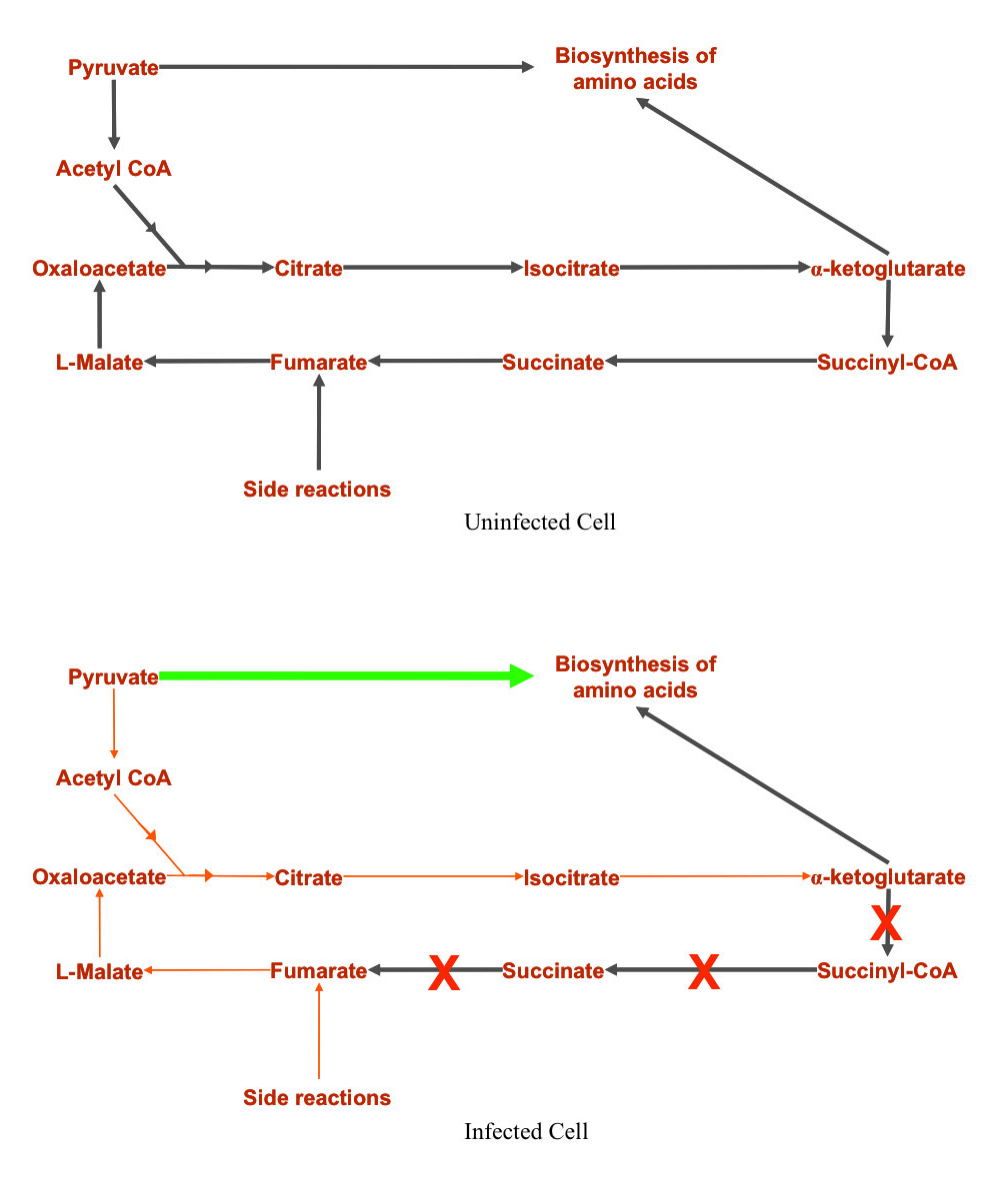
**Comparison of TCA cycle of an uninfected and a MS2 phage-infected *Escherichia coli *cell**. Thick flux arrows represent high reaction fluxes, whereas the thinner flux arrows represent low reaction fluxes. Biosynthesis of amino acids is upregulated in infected cells leading to the diversion of pyruvate to the biosynthesis of amino acids. As a result, the conversion of pyruvate to acetyl-CoA is downregulated. This severely downregulates the TCA cycle. The amount of α-ketoglutarate formed is just enough to act as the pre-cursor for the biosynthesis of amino acids. This is where the TCA cycle breaks leading to the deactivation of the reactions downstream (represented by red crosses). The activity of the last two reactions in the TCA cycle is only due to the formation of fumarate as a by-product of other reactions. Even so, these reactions are markedly downregulated.

The increase in biosynthesis of amino acids also has an effect on the TCA cycle, as shown in Figure 6. In an uninfected cell, pyruvate is converted to acetyl-CoA that in-turn feeds the TCA cycle. Pyruvate and α-ketoglutarate also act as pre-cursors for the biosynthesis of amino acids. In infected cells, the up-regulation of amino acid biosynthesis leads to the diversion of a major part of pyruvate to the biosynthesis process. As a result, smaller amounts of acetyl-CoA are formed leading to the severe down-regulation of the TCA cycle. The amount of α-ketoglutarate formed is inadequate and just enough to act as the pre-cursor for the biosynthesis of amino acids but not enough to resume as a part of the TCA cycle. It is here that the TCA cycle breaks down leading to the deactivation of the reactions downstream. The activity of the last two reactions, FUM and MDH, in the TCA cycle is only due to the formation of fumarate as a by-product of other reactions. Even so, these reactions are markedly downregulated.

## Conclusions

A systems biological approach was taken to investigate the effects of viral infection on the host metabolism. It was seen that the viral infection alters the host metabolism to upregulate the biosynthesis of amino acids. Resources were diverted away from the TCA cycle and from the synthesis of cell wall components in order to upregulate the pentose phosphate pathway. This finally resulted in the production of viral proteins. Interestingly, a similar phenomenon has been noted in cancer cells where glucose is used primarily for anabolic processes via the pentose phosphate pathway [32,33], suggesting the use of the *E. coli/*MS2 system as a potential test bed for the study of drugs targeting metabolism for the treatment of cancer. Clearly, however, any such approach would require far more rigorous study of the commonalities between the viral infection process and cancer metabolism before any real parallels can be drawn.

This study is a proof of principle where techniques of modeling the metabolic system of a virus-infected host were described. Incorporation of regulated viral gene expression, viral genome replication and a suitable objective function to model an infected cell were shown. The greatest advantage of this approach is that the effects can be seen at full genome-scales. Advances in both experimental and computational fields could be used to extend this work to higher order organisms. Efforts to model human cellular metabolism are taking place [34-36] and application of the methods described in the current study may give new insights. Information based on the up-regulation and down-regulation of certain reactions could then be utilized to draw new treatment strategies.

## Authors' contributions

RJ and RS conceived and designed the study. RJ carried out the experiments and implemented the models. All authors read and approved the final manuscript.

## Supplementary Material

Additional file 1 **Calculation of uninfected and infected cell properties**. Experimental data provided was used to calculate various properties of uninfected and MS2-infected *Escherichia coli *cells. Click here for file
